# Hemangiopericytoma/solitary fibrous tumor of the cranial base: a case series and literature review

**DOI:** 10.1186/s12893-022-01718-5

**Published:** 2022-07-27

**Authors:** Zhouying Peng, Yumin Wang, Yaxuan Wang, Qinxuan Li, Yan Fang, Ruohao Fan, Hua Zhang, Weihong Jiang

**Affiliations:** 1 Department of Otolaryngology Head and Neck Surgery, Xiangya Hospital, Central South University, Changsha, 410008 Hunan China; 2 Otolaryngology Major Disease Research Key Laboratory of Hunan Province, Changsha, 410008 Hunan China; 3 National Clinical Research Center for Geriatric Disorders, Xiangya Hospital, Central South University, Changsha, 410008 Hunan China; 4 Anatomy Laboratory of Division of Nose and Cranial Base, Clinical Anatomy Center of Xiangya Hospital, Central South University, Changsha, 410008 Hunan China

**Keywords:** Hemangiopericytoma, Solitary fibrous tumor, Cranial base, Case series, Survival

## Abstract

**Background:**

Hemangiopericytomas (HPCs) are uncommon soft tissue tumors. HPCs that grow in the cranial base are rare. Therefore, skull-base surgeons tend to overlook this disease. This study aimed to increase the awareness of HPCs by summarizing case data from our institution and related publications. We also aimed to contribute to the number of reported cases for future systematic reviews of HPCs.

**Methods:**

This study included all patients who underwent surgery for HPC/solitary fibrous tumor (SFT) between August 2015 and August 2019. All surgeries were performed at Xiangya Hospital Central South University. We analyzed clinical characteristics, surgical highlights, treatment modalities, and outcomes.

**Results:**

We included six patients, aged 32–64 years. Lesions were located in the parapharyngeal space in three patients, pterygopalatine fossa in two, and saddle area in one. All patients underwent nasal endoscopic endonasal surgery. In five patients, tumors involved the internal carotid artery (ICA). The exposure and protection of the ICA during surgery are challenging but critical to complete tumor removal. The 3-year overall survival(OS) rate was 66.7%.

**Conclusions:**

HPC/SFTs are rare tumors of the cranial base that are prone to recurrence. Cranial base HPC/SFTs are often closely associated with the ICA. To our knowledge, this case series reports the largest number of cases of HPCs associated with the ICA. We believe that there is a strong relationship between patient prognosis and whether the tumor encircles the ICA and whether the tumor is completely resected. To confirm this suggestion, more cases are needed for further analysis.

**Supplementary Information:**

The online version contains supplementary material available at 10.1186/s12893-022-01718-5.

## Introduction

Hemangiopericytoma (HPC), also known as solitary fibrous tumor(SFT), were first described by Stout and Murray. HPCs may be benign or malignant and originate from the pericytes of the capillary and venous walls [1]. Pathologically, HPCs are characterized by abundant spindle cells and “staghorn” vascular branches. HPCs can occur anywhere in the body, with the most common locations being the trunk, pelvis, and lower extremities. Of all reported cases, 15–25% occurred in the head and neck. Only 5% have been found in the nose or paranasal sinuses [2, 3]. HPCs are found in the cranial base region. Epidemiologically, there is no sex, age, or ethnic predilection for the occurrence of HPCs/SFTs. HPCs/SFTs are clinically characterized by malignancy-like aggressiveness, a tendency to recur, and metastasis risk [4, 5]. Surgery remains the mainstay of treatment. Of note, given the rarity of the diagnosis, it is difficult to assess the impact of radiotherapy or chemotherapy [6]. Due to the paucity of studies on this condition, the factors affecting its prognosis are not clear, but total intraoperative tumor resection is important. Existing publications on cranial base HPCs/SFTs are few and mostly case reports. Hence, only limited assumptions can be made about the tumor.

We collected data on patients with cranial base HPCs/SFTs who received surgical treatment at our institution in the last 4 years. We also summarized the findings from available publications including the clinical features and treatment. These details were combined to complement the small but growing literature on HPCs. We aimed to share our experiences and lessons learned in the treatment of HPCs, which are closely related to the internal carotid artery (ICA). Our insights may be useful to cranial base surgeons for the diagnosis and treatment of this type of tumor.

## Materials and methods

 We retrospectively reviewed the data of patients who underwent surgical treatment for cranial base tumors from August 2015 to August 2019 and summarized the details, including the results of the pathological examinations. Consent was obtained from the patient or the patient’s family. The study was approved by our institutional ethics department.

Imaging examinations, such as cranial base computed tomography (CT), cranial base enhanced magnetic resonance imaging (MRI), cranial base magnetic resonance angiography (MRA), or cranial base digital subtraction angiography, to show the tumor and its relationship with important anatomical structures were performed for all patients. All endoscopic endonasal surgeries (EES) were performed by Dr. Weihong Jiang at Xiangya Hospital Central South University. Additional clinical information was collected through medical case records and at follow-ups.

We summarized the key points relating to the exposure and protection of the ICA during tumor resection surgeries by analyzing the surgical videos. We also compared the patient’s preoperative and postoperative cranial base MRI findings to determine whether the tumor had been fully removed. We analyzed the overall survival (OS) rate of these patients using the Kaplan–Meier method in SPSS (version 21.0, SPSS Inc., Chicago, IL, USA).

We also summarized the available publications on cranial base HPCs/SFTs and combined the details with our patient data. Furthermore, we discussed the key points of diagnosis and treatment and analyze the factors affecting prognosis.

## Results

### Clinical characteristics of included patients

Our study included six patients (three men and three women), aged 32–64 years. Lesions were located in the parapharyngeal space in three patients, pterygopalatine fossa in two, and saddle area in one. The investigated tumor characteristics included the tumor topographical site, pathological type, differentiation status, and pathological stage. Patient characteristics are shown in Table 1.

**Table 1:** Demographics and clinical characteristics of cranial base hemangiopericytoma/solitary fibrous tumor

Patient No.	Gender	Age (years)	Localization	Clinical manifestation	Previous treatment	Relationship to the ICA	Post-operative treatments	Follow-up time (months)	Survival ending
#1	Male	50	Centered on the right PF, involving the PA, FL, and CS	FP, difficulty opening mouth, diplopia	Three times surgeries, four times gamma treatments	Encircled	None	16	Died
#2	Male	49	Left parapharyngeal space, ET	HL, epistaxis	None	Encircled	None	70	Alive
#3	Female	47	Right SA	Vision loss, diplopia	One time surgery	Encircled	Right ICA embolization	48	Alive
#4	Female	32	Centered on the right parapharyngeal space, involving the right middle CF and CS	FP	None	Encircled	Radiotherapy	48	Alive
#5	Male	64	Right parapharyngeal space	FP, headache, dysphagia	Four times surgeries	Encircled	None	24	Died
#6	Female	53	Left NC and PF	Epistaxis, nasal obstruction, HL	None	None	None	24	Alive

PF: pterygopalatine fossa; PA: petrous apex; FL: foramen lacerum; CS: cavernous sinus; SA: saddle area; CF: cranial fossa; NC: nasal cavity; FP: facial paralysis; HL: hearing loss; ICA: internal carotid artery; ET: eustachian tube

### Treatment and prognosis of included patients

**Fig. 1:**
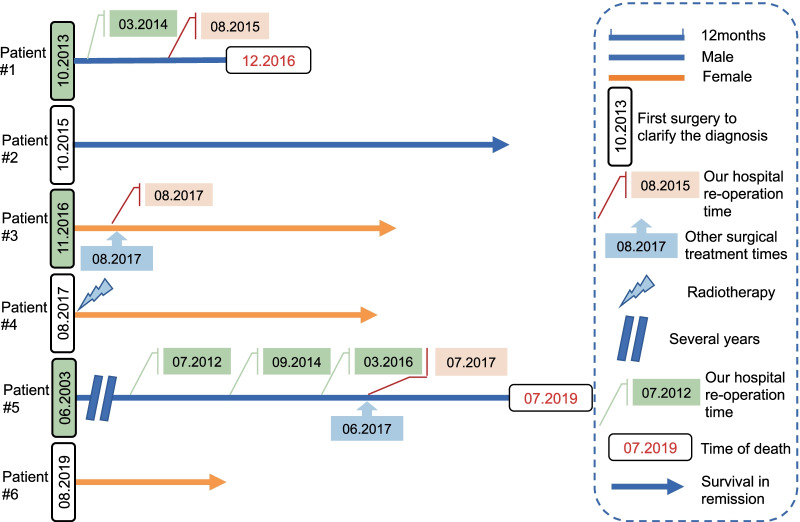
Swimming-plot shows the treatment process for each patient

**Fig. 2:**
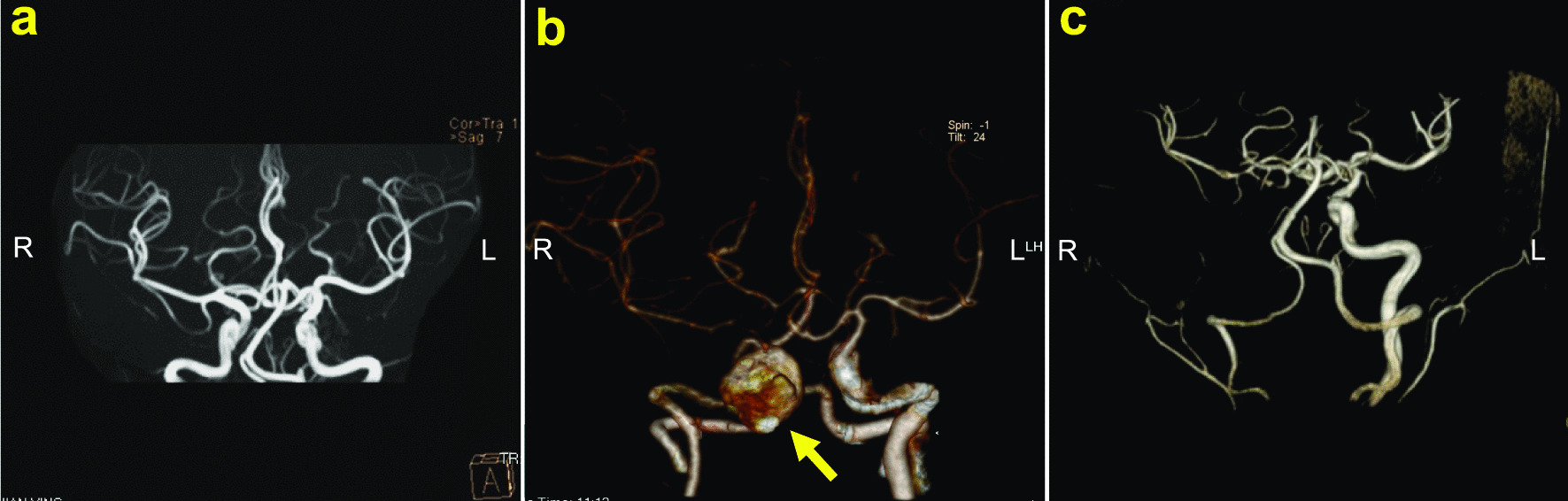
Patient #3’s pre- and postoperative imaging of ICA balloon embolization. **a**, **b** he preoperative imaging, yellow arrow indicates the pseudoaneurysm. **c** The post-operative imaging

Three patients underwent their first surgery in other hospitals. Patients #1 and #5 underwent multiple surgical procedures. Only one patient received radiotherapy after the last surgery. The specific consultation processes and outcomes are shown in Fig. 1. Patient #3 was found to have a pseudoaneurysm on postoperative MRA. As a result, a balloon embolization of the ICA was performed (Fig. 2).

**Fig. 3:**
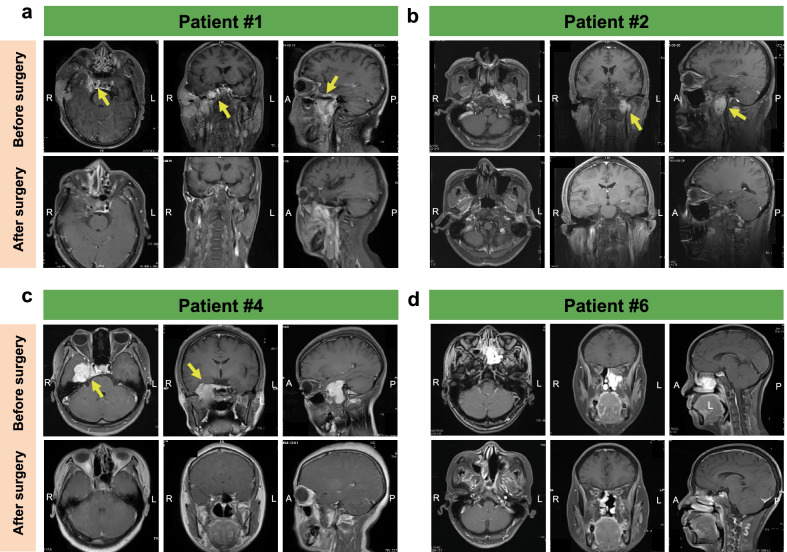
Preoperative and postoperative MRI images of the cranial base. **a** Patient #1. **b** Patient #2. **c** Patient #4. **d** Patient #6

The average follow-up time was 36 months (range, 16–70 months). At the time of writing, four patients were still alive. The main cause of death was cachexia. The 3-year OS rate was 66.7%. Figure 3 shows patients with preoperative to postoperative imaging comparisons. Patients #3 and #5 had mismatched pre- and postoperative imaging because they were primarily reviewed at other hospitals postoperatively. The images of patients #3 and #5 are shown in Additional file 1: Fig. S1. None of the patients had new neurological deficits after the final surgery.

### Visualization and protection of ICA during EES

**Fig. 4:**
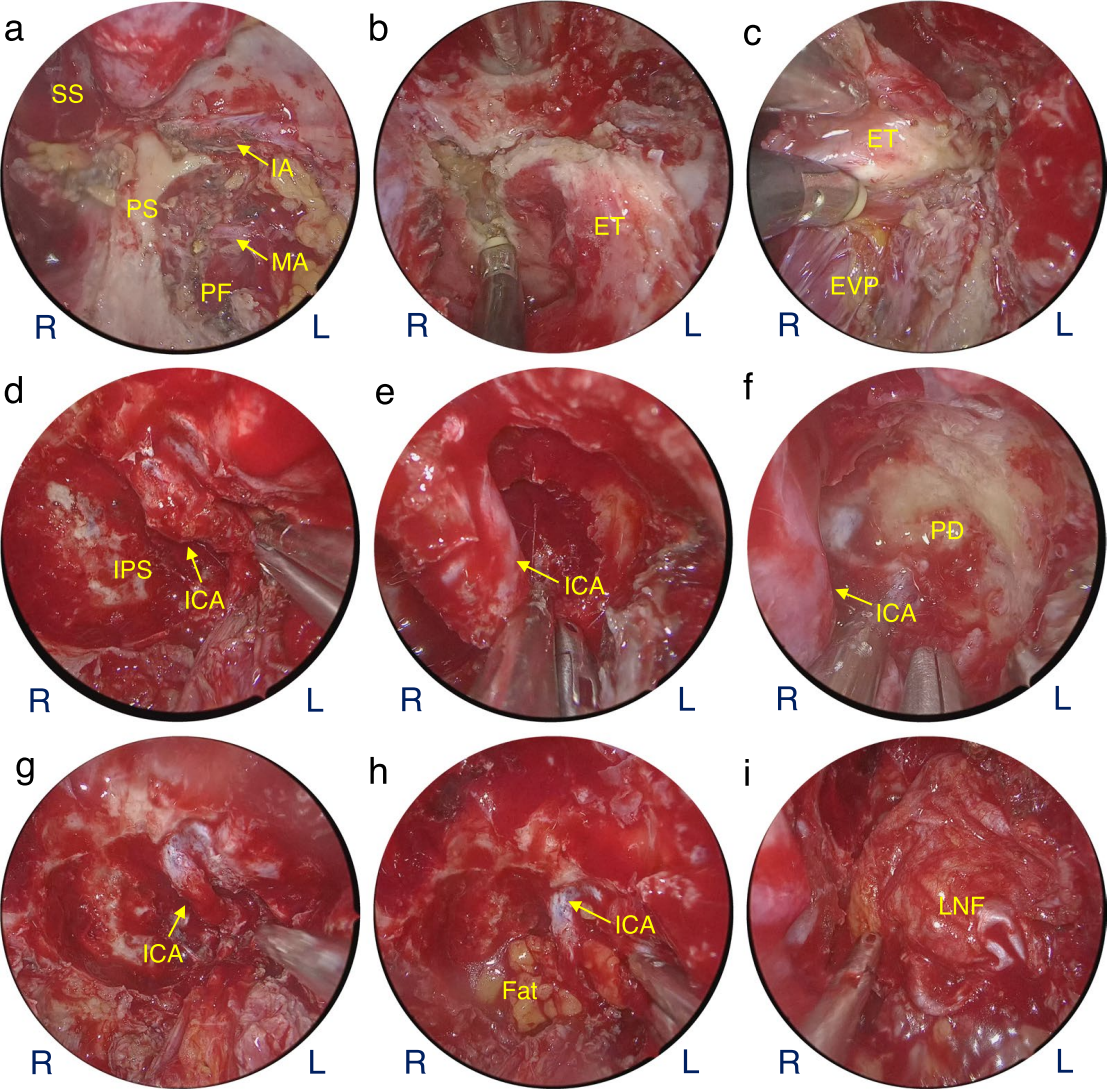
Exposure and protection of ICA in endoscopic endonasal surgery. **a** Reveal the contents of the PF. **b**, **c** Removal of the ET and preservation of the EVP. **d–g** Removal of lesions around ICA, free ICA. **h** Fill in autologous fat. **i** Reconstruction of cranial base with LNF. SS, sphenoid sinus; PS: pterygoid process; IA: infraorbital artery; MA: maxillary artery; PF: pterygopalatine fossa; ET: eustachian tube; EVP: evator veli palatine; IPS: inferior petrosal sinus; ICA: internal carotid artery; PD: petrous drum; LNF: lateral nasal flap

In five of the six patients in this group, the tumor was closely related to the ICA of the cranial base segment. Patient #2 is used as an example to illustrate the importance of revealing and protecting the ICA in the EES of cranial base HPCs. Figure 4 shows the process of resecting the lesion around the ICA. The resection of the cranial base HPCs was performed in two parts: resection of the lesion and reconstruction of the cranial base structures. Patient #2 had a lesion that encircled the ICA from the paraclival to the parapharyngeal space, so safe resection of the tumor surrounding the ICA was critical to the success of the surgery. Using the pterygoid process(PS) as a landmark to reveal the eustachian tube(ET) and the foramen lacerum(FL). Then resection of the ET while preserving the evator veli palatine can guide the search for an ICA. After removal of the lesion surrounding the ICA (Figure d–f), the adventitia of the ICA is removed, as in Figure g. After the ICA was released from the paraclival to the parapharyngeal space, autologous fat was filled to prevent ICA suspension, and the skull base was repaired with an artificial dura and a pre-prepared lateral nasal wall flap.

#### Pathology of included patients

The pathology results of the cases in this group all showed hemangiopericytomas (Grade II), and immunohistochemical tests were performed in all cases (Table 2). Among the six included patients, only patient #4 (a young female patient) was treated in the oncology department for postoperative intensity-modulated radiotherapy supplemented with radiotherapy for sensitization, while the rest of the patients did not receive radiotherapy or other combination therapy after surgery.

**Table 2:** Details of the pathological examination of Cranial Base Hemangiopericytoma/ Solitary Fibrous Tumor

Patients No.	WHO classification	Immunohistochemical results
#1	II	SSTR2A(−), STAT6(+), CD34(-), Ki67(~ 5%+)
#2	II	CD34(+), STAT6(+), Ki67(~ 5%+), CD99(+), SSTR2A(−)
#3	II	CD34(−), Bcl-2(+), CD99(+), STAT6(+), SSTR2A(-),PR(-), Ki67(1%+)
#4	II	CD34(+), STAT6(+), Ki67(3%+), PR(−), CD99(+), Bcl-2(+), SSTR2A(−)
#5	II	CD34(++), Bcl-2(+), STAT6(+), SSTR2A(-), Ki67(15%+), CD99(−)
#6	II	CD34(part+), STAT6(+), SSTR2A(−), CgA(−), Syn(+), Ki67(5%+), EMA(−), E-cadherin(−), PR(part+), CD31(+), F8(-), NeuN(−), NF-Pan(-)

The pathology results showed hemangiopericytomas (grade II) for all patients. Immunohistochemical tests were performed (Table 2). Only patient #4 (a young female patient) was treated in the oncology department for postoperative intensity-modulated radiotherapy, supplemented with radiotherapy for sensitization. The other patients did not receive radiotherapy or other combination therapy after surgery.

## Discussion

HPCs/SFTs account for less than 1% of all vascular tumors [7]. Fewer than 5% occur in the nose or sinuses. HPCs of the skull base are extremely rare. HPCs/SFTs exhibit malignant features and are prone to recurrence and metastasis [4–6]. Despite the low incidence of this tumor, the specific nature of the condition makes the diagnosis and treatment of HPCs/SFTs of the cranial base difficult. Previous publications on HPCs were mostly case reports. Our efficacy results were not better than those of previous studies. Therefore, we have summarized our cases and findings from the literature.

### Diagnosis of cranial base HPCs/SFTs

**Fig. 5:**
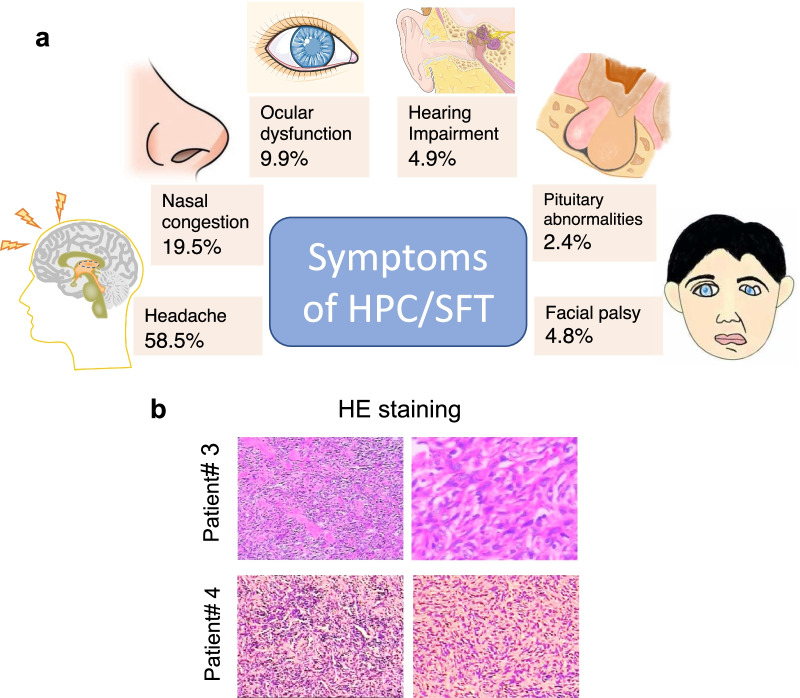
a Shows the summarization of clinical symptoms of patients with cranial base HPC/SFT from currently available publications. **b** Shows the HE staining picture of the typical HPCs’ patient

The clinical presentation of patients with HPCs/SFTs of the cranial base may vary depending on the specific site of growth. If the main body of the tumor is located in the nasal cavity and grows toward the cranial base, nasal symptoms, such as nasal congestion and epistaxis, tend to be the main clinical manifestations, and the patient may not report any headaches and earaches. If the main body of the tumor is in the anterior skull base, infratemporal fossa, pterygopalatine fossa, or parapharyngeal space, headaches are most common. Corresponding regional neurological dysfunction, such as distorted mouth, hearing loss, and vision loss, and other symptoms may also be evident. From the literature, headache symptoms were predominant (24/41), followed by ocular symptoms (4/41), auditory symptoms (2/41), facial palsy symptoms (2/41), nasal symptoms (8/41), and pituitary abnormalities (1/41) (Fig. 5A) [8–20]. Patients often undergo ENT-head and neck surgery or neurosurgery for these clinical complaints. HPCs/SFTs rare, and the lesions have nonspecific clinical features. Hence, it is often difficult to define the pathology preoperatively. Imaging is a valuable tool in the diagnosis of HPCs. Moreover, enhanced cranial base CT and MRI are the preferred imaging techniques [11]. Features of HPCs/SFTs include an isolated mass with relatively well-defined irregular or dumbbell-shaped borders, which may have internal signal voids. On CT, the tumor often shows a heterogeneous enhancing signal shadow, with or without bone destruction.

The gold standard for the diagnosis of HPCs/SFTs of the cranial base remains pathological diagnosis [10], and preoperative biopsy is still necessary for those whose lesion main location in the nasal cavity or whose cranial base lesion invade the nasal cavity. The pathological findings of our cases were synthesized, and some pathological features of perivascular cell tumors were summarized in conjunction with literature reports. Under light microscopy, oval and spindle-shaped cells can be seen, with vessels ranging in size from tiny capillary-like slits to larger open channels, and a typical antler-like pattern of vessels is usually seen [11]. In contrast, immunohistochemical findings are helpful in the differential diagnosis of this disease and other tumors such as meningioma; we synthesized the immunohistochemical findings of previous literature on HPCs/SFTs of the cranial base and combined them with our cases and found that the immunohistochemical findings of HPCs/SFTs are usually CD34 (n = 35/45, 77.8%), Vimentin (n = 10/11,90.9%), Bal-2 ( n = 8/9,88.9%), STAT6 (n = 28/29,96.6%), and Ki67 index ranging from 8 to 40%. In contrast, S100 (n = 3/7,42.9%), CD31 (n = 2/9,22.2%), and EMA (n = 0/15) were largely unexpressed [8, 9, 12, 15, 18, 21–27]. CD34 (+) and EMA (−) can better distinguish HPCs/SFTs from meningiomas that are more similar in histomorphology [15, 28], Fig. 5B shows a typical HE staining picture of HPC.

### Treatment of cranial base HPCs/SFTs

HPCs/SFTs have pro-vascular characteristics [29]. The ICA often encircles or becomes invaded by the tumor. Hence, the protection of the ICA during surgical treatment of cranial base lesions is particularly important. For surgical treatment of HPCs/SFTs, it is crucial to achieve total dissection of the ICA as much as possible. However, owing to the specificity of the location of the lesion, surgical excision of the lesion without damaging important vascular and neurological functions requires a high level of anatomical knowledge of the cranial base and technique sensitivity. During the resection of giant HPCs/SFTs at the cranial base, if it is not possible to remove the tumor completely, staged surgery to protect nerve function and avoid damage to important blood vessels should be considered.

HPCs/SFTs are usually aggressive, and the probability of recurrence and distant metastasis is not low. Therefore, postoperative radiotherapy is necessary to prevent tumor recurrence [30]. Although only one patient in the present study received postoperative radiotherapy, the other patients, who did not receive radiotherapy, did not experience recurrence. Based on only a few cases, it is not possible to determine the necessity of postoperative radiotherapy and other comprehensive treatments. Whether postoperative radiotherapy has any effect on improving survival requires further studies with larger cohort analyses.

### Visualization and protection of the ICA during EES

In cranial-based HPCs, ICA disclosure and protection are critical to the success of total tumor resection in EES. Many cranial base HPCs are closely related to the ICA of the skull base segment, and the tumor is often adjacent to or encircling the ICA. Patrona et al. reported a case of HPC involving the cavernous carotid artery (CCA) and discussed several skull base tumors involving the cavernous sinus region [17]. Five of our patients had HPCs involving the skull base segment of the ICA, CCA, ruptured segment of the ICA, and horizontal and vertical segments of the ICA. The parapharyngeal space segment was predominant. The need for ICA embolization on the side of the lesion before EES was considered according to the involvement of different ICA segments and the BOT test results.

### Postoperative management of cranial base HPCs/SFTs

Due to the specificity of the site and growth pattern of cranial base HPCs/SFTs, the these lesions often grow around important blood vessels or nerves. In five of six patients in this study, the ICA was encircled by the tumor, which greatly increased the difficulty of the surgery and the risk of postoperative intracranial hemorrhage and other accidents. In patient #3, a pseudoaneurysm of the ophthalmic segment of the ICA appeared after surgery. For patients whose imaging shows a close relationship between the tumor and the blood vessels, CTA or MRA should be routinely performed before surgery. Moreover, the above tests should be reviewed after surgery to prevent and exclude the occurrence of aneurysms and other conditions [31]. For patients with lesions located in specific functional areas of the skull base, such as tumors in the saddle area that are closely related to the pituitary gland, preoperative and postoperative pituitary function tests should be performed to monitor pituitary function, so that postoperative symptoms can be detected and treated promptly.

Our goal was to share our experience in the diagnosis and treatment of HPCs, especially the importance of intraoperative visualization and protection of the ICA. We also aimed to contribute to the knowledge of this disease for cranial base surgeons.

## Conclusions

HPC/SFTs are rare tumors of the cranial base that are prone to recurrence. Cranial base HPC/SFTs are often closely associated with the ICA. To our knowledge, case series reports the largest number of cases of HPCs associated with the ICA. We believe that there is a strong relationship between patient prognosis and whether t the tumor encircles the ICA and whether the tumor is completely resected. To confirm this suggestion, more cases are needed for further analysis.

## Supplementary Information

**Additional file 1: Figure S1.** Preoperative and postoperative MRI images of thecranial base. (a) Patient #3 (b) Patient #5.

## Data Availability

All data generated or analysed during this study are included in this published article and its supplementary information files.

## Abbreviations

HPCs: Hemangiopericytomas
SFT: Solitary fibrous tumor
PS: Pterygoid process
PF: Pterygopalatine fossa
SA: Saddle area
EES: Endoscopic endonasal surgeries
ICA: Internal carotid artery
CCA: Cavernous carotid artery
CT: Computed tomography
MRI: Magnetic resonance imaging
MRA: Magnetic resonance angiography
DSA: Digital subtraction angiography
PA: Petrous apex
FL: Foramen lacerum
CS: Cavernous sinus
CF: Cranial fossa
NC: Nasal cavity
FP: Facial paralysis
HL: Hearing loss
ET: Eustachian tube
MA: Maxillary artery
IA: Infraorbital artery
SS: Sphenoid sinus
EVP: Evator veli palatine
PD: Petrous drum
LNF: Lateral nasal flap
IPS: Inferior petrosal sinus
